# Needle Decompression of Tension Pneumoperitoneum: A Case Report

**DOI:** 10.5811/cpcem.2021.8.53447

**Published:** 2021-11-01

**Authors:** Joseph Ray, Nadin Exantus

**Affiliations:** AdventHealth East Orlando, Department of Emergency Medicine, Orlando, Florida

**Keywords:** tension pneumoperitoneum, needle decompression, overdose, gastric perforation

## Abstract

**Introduction:**

Tension pneumoperitoneum is rarely encountered in the emergency department but can have disastrous effects on the body when it is. However, an emergency physician has skills that can be readily applied to needle decompress the abdomen for rapid stabilization.

**Case Report:**

A 42-year-old male arrived via ambulance after a likely overdose with mental status improvement following naloxone administration. He was found to be in respiratory distress due to a rigid, distended abdomen that required intubation for stabilization. Computed tomography imaging showed significant pneumoperitoneum with tension physiology. Surgery consultation was unable to intervene immediately, and needle decompression with an angiocatheter was performed at the bedside with immediate ventilatory improvement.

**Conclusion:**

Tension pneumoperitoneum is a rare but potentially disastrous consequence of overdose secondary to emesis and rupture of the gastric wall. Needle decompression is a skillset already in the emergency physician’s toolbox and can be applied for emergency stabilization of a tension pneumoperitoneum with proper forethought and technique.

## INTRODUCTION

Tension pneumoperitoneum is a rare disease process that few will encounter in the emergency department (ED) setting. While frequently an unexpected adverse event of endoscopic procedures, any perforation of an abdominal hollow viscus can develop into a tension pneumoperitoneum in the right setting.^1^ Although definitive management to correct the hollow viscus injury remains surgical, intervention by the emergency physician is warranted in the setting of unstable vital signs or prolonged delay in transfer to the operating room. This case illustrates the use of bedside needle decompression for immediate tension relief and restoration of abdominal organ perfusion prior to operative internal repair.

## CASE REPORT

A 42-year-old male presented to the ED via emergency medical services (EMS) after being found unconscious on his porch by a neighbor. The EMS team found him minimally responsive with pinpoint pupils and emesis nearby. He was given 2 milligrams (mg) of intranasal naloxone followed by 6 mg of intravenous (IV) naloxone by EMS during transport and regained consciousness. Initially oxygen saturation was 72% and he was placed on 12 liters of supplemental oxygen via a non-rebreather with improvement to 88% by arrival to the ED.

Upon arrival, the patient was awake and noted that he had no past medical history and was not on any prescription medications. He stated he had taken a handful of alprazolam for recreational purposes earlier in the day. His only complaints were significant shortness of breath and mild diffuse abdominal pain. Initial vital signs were heart rate of 154 beats per minute, respiratory rate of 45 breaths per minute, oxygen saturation of 93% on 12 liters per minute oxygen by non-rebreather. Physical examination showed a male with vomitus on his shirt. He was tachypneic with shallow labored breathing without crackles or wheezing. His abdomen was distended and tense with mild diffuse tenderness to palpation and no external signs of trauma. He was awake and responding appropriately to questions but only in two- to three-word phrases secondary to dyspnea. Remaining physical exam was unremarkable, including peripheral pulses, pupils, and motor function.

**Image 1:**
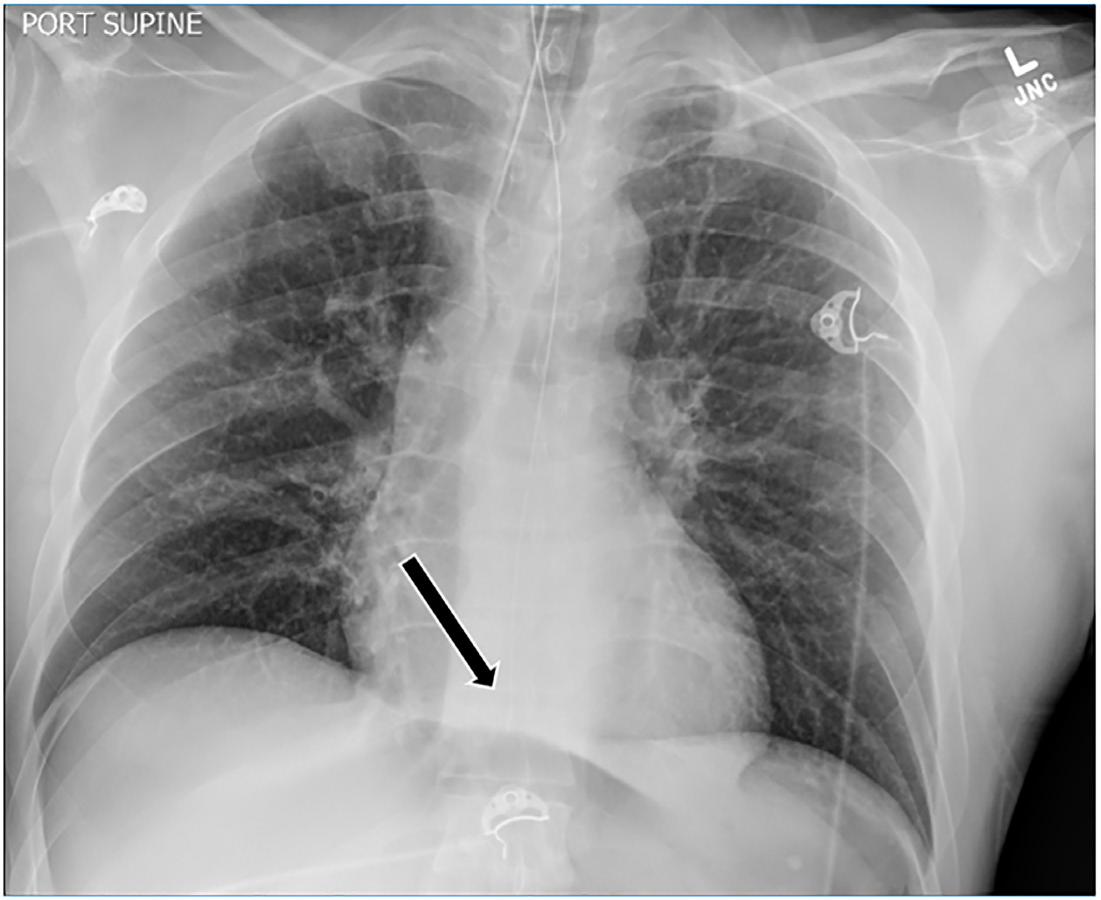
Post-intubation radiograph showing endotracheal tube placement and orogastric tube just superior to the level of the diaphragm, as shown by arrow.

The patient’s respiratory status continued to deteriorate and he was emergently intubated. His oxygen saturation immediately improved to 98% on the ventilator. A single dose of rocuronium (50 mg) was given for ventilator dyssynchrony, despite the increasing propofol infusion that was initiated for continued and post-intubation sedation. His abdomen remained distended and firm despite orogastric (OG) tube, Foley catheter, and improving respiratory status. Post-intubation chest radiograph (Image 1) showed an OG tube at the level of the diaphragm, a correctly placed endotracheal tube, and no identifiable pathology.

With his improved vital signs and increased stability post intubation, the patient was sent for a computed tomography of the abdomen. Point-of-care labs drawn prior to intubation showed significant values of pH 7.12 (reference range: 7.31–7.41) and partial pressure of carbon dioxide of 61.3 millimeters of mercury (mm Hg) (45–50 mm Hg) on venous blood gas, creatinine of 1.7 milligrams per deciliter (mg/dL) (0.6–1.2 mg/dL), and lactic acid of 5.5 millimoles per liter (mmol/L) (0.5–1.9 mmol/L). Other laboratory results were mostly unremarkable but included white blood cell count of 12.2 thousand/microliter (uL) (4.4–10.5 thousand/uL), glucose of 332 mg/dL (70–100mg/dL), and mildly elevated transaminases. The patient was given vancomycin, cefepime, and IV fluids for presumed severe sepsis.

**CPC-EM Capsule**

What do we already know about this clinical entity?

*High intraperitoneal pressures do cause immediate life threatening distress and require emergent intervention*

What makes this presentation of disease reportable?

*This entity is most frequently encountered in a surgical setting and managed via operative intervention – leaving emergency medicine physicians less experienced with management*

What is the major learning point?

*Needle decompression, a technique we are well accustomed to regarding the thorax, can also be applied for temporary stabilization of tension pneumoperitoneum.*

How might this improve emergency medicine practice?

*Further heightening suspicion for this disease process and educating on emergency department management.*

**Image 2:**
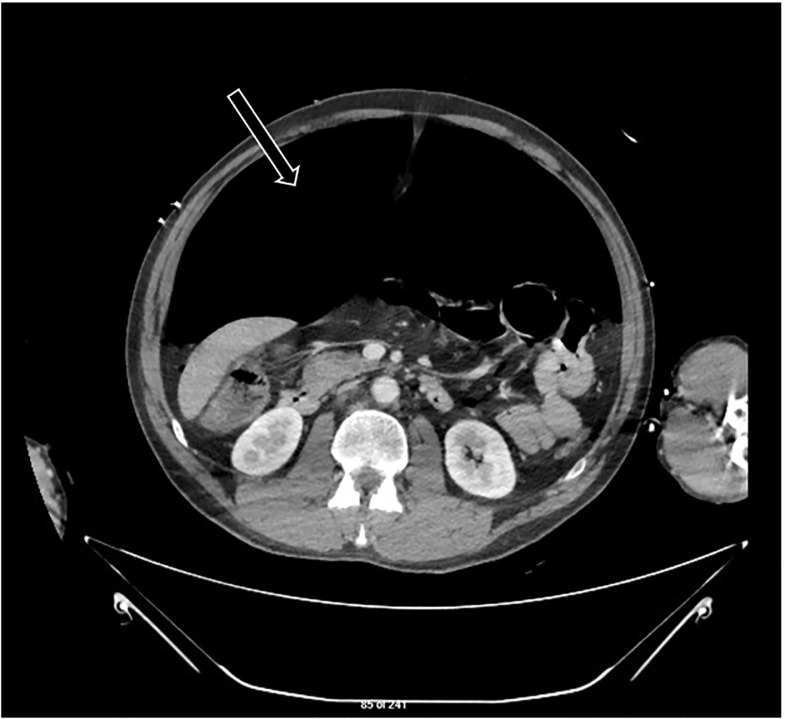
Computed tomography of the abdomen with intravenous contrast at the level of the inferior tip of the liver and kidneys showing large pneumoperitoneum, as noted by arrow, with tension physiology.

Computed tomography of the abdomen revealed a large amount of pneumoperitoneum creating tension physiology in the abdominal compartment (Image 2), and general surgery was consulted. Given the emergent nature of the condition, the temporal limitation with the operating room, and surgical staff availability, it was decided that the ED team would needle decompress the abdomen while the operating room was being assembled.

The patient was placed in a supine position and his abdomen sterilized with chlorhexidine. A 14 French angiocath was inserted into the anterior right lower quadrant of the abdomen until a rush of air returned. At that point, the catheter was advanced and the needle removed while air continued to flow from the peritoneal cavity. Once the abdomen was soft, a Luer lock was placed on the angiocath and the angiocath secured with an occlusive dressing. After decompression, ventilator dyssynchrony significantly improved.

**Image 3:**
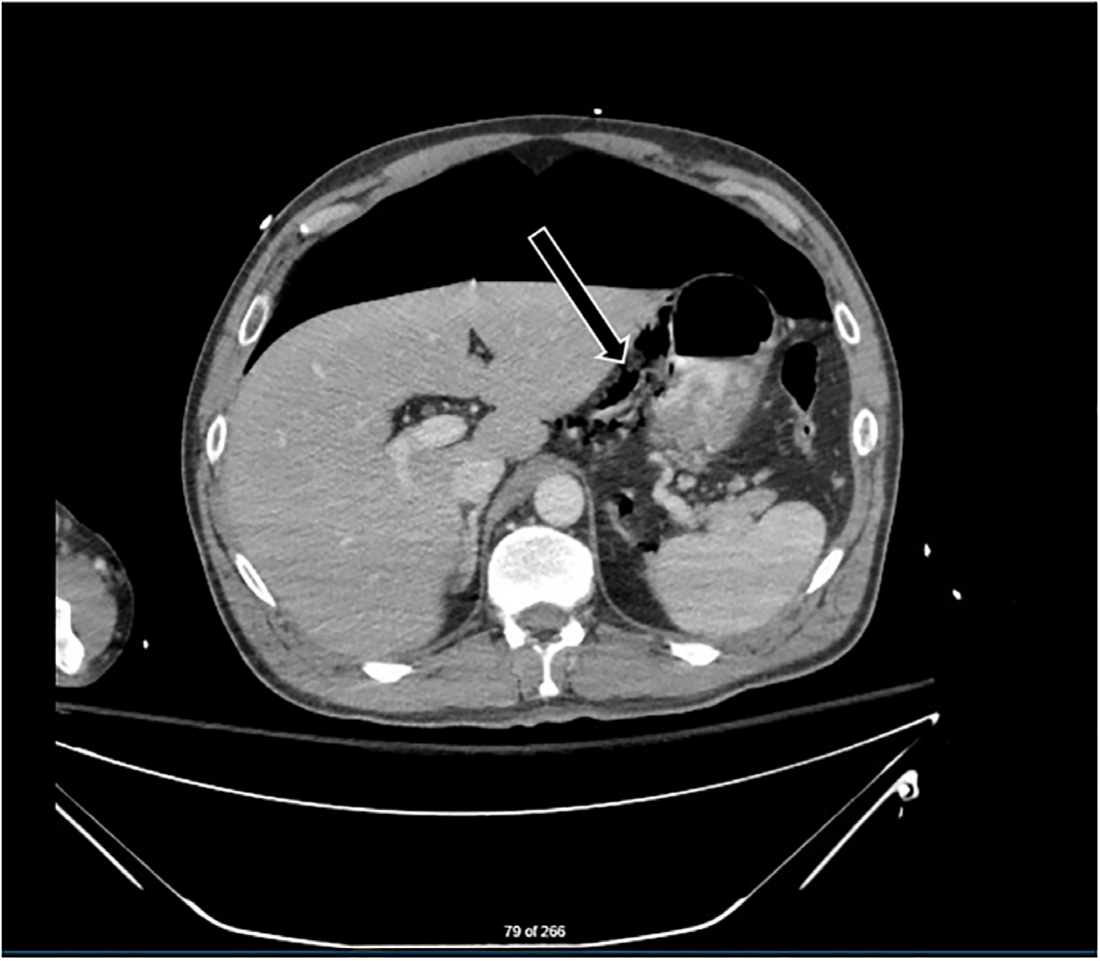
Computed tomography of the abdomen and pelvis with oral contrast at the level of the stomach performed after needle decompression. Large pneumoperitoneum was still present but without tension. Arrow points to air bubbles seen along lesser gastric curvature suggesting location of perforation.

With the patient stabilized, the general surgeon requested a CT of the abdomen and pelvis with oral contrast for further localization of the injury. Due to concerns for perforation, the decision was made to not advance the previously short OG tube any farther but to continue to use it to instill water-soluble contrast into the gastrointestinal tract. While the repeat imaging was not definitive, the likely site of viscous perforation was thought to be the lesser curvature of the stomach (Image 3).

Ultimately the patient was found to have a 1-centimeter (cm) perforation of the lesser curvature of the stomach, which was surgically repaired. He was subsequently admitted to the intensive care unit (ICU) following surgery and discharged home on hospital day seven.

## DISCUSSION

Few cases of tension pneumoperitoneum have been reported in the medical literature; most are the result of either endoscopic procedures or trauma.^1^–^3^ Cardiopulmonary resuscitation (CPR) with rib fracture perforating the stomach has also been noted as a cause of tension pneumoperitoneum.^4^ However, the case described here had neither endoscopy nor trauma, instead originating from a spontaneous gastric perforation in conjunction with overdose and intense vomiting.

Tension pneumoperitoneum may be compared to abdominal compartment syndrome in clinical presentation.^5^ This may present with abdominal distention, poor abdominal organ perfusion, and resulting elevated lactic acid levels.^6^ The high abdominal compartment pressures will cause splinting of the diaphragm, preventing adequate ventilation, as well as compression of the vena cava, thus decreasing venous return and consequently cardiac output.^6^ Patients will likely be critically ill and require immediate stabilization. Our patient required mechanical ventilation due to the diaphragmatic splinting as part of initial stabilization, but cases resulting from trauma or CPR may have multiple concomitant injuries, such as tension pneumothorax,^3^ contributing to respiratory failure.

As with tension pneumothorax, needle decompression can provide temporary stabilization by reducing intracompartment pressures. While tension pneumoperitoneum has been described in the surgical literature after endoscopic procedures or abdominal trauma, it has not been well documented as a result of a spontaneous gastric perforation. Furthermore, cases that are documented have been frequently managed by either needle decompression in a surgical ICU or with emergent laparotomy. The case described here is novel for both being the result of a spontaneous gastric perforation and for requiring the needle decompression intervention to be performed by an emergency physician.

While no standard of care has been established on how to perform such a procedure, our technique with a readily available angiocath was highly effective and easy to perform. This technique has been used in surgical services for iatrogenic bowel perforation causing tension pneumoperitoneum but can also be applied in the ED setting.^7^ Consideration of angiocath insertion under ED bedside ultrasound guidance should be considered when attempting to mitigate the risk of further viscous injury. Just as would be the case in an abdominal paracentesis, potential complications include failed procedure, abdominal wall hematoma, spontaneous hemoperitoneum due to mesenteric bleeding, hollow viscus perforation, catheter loss in the abdominal cavity, and major blood vessel laceration.^8^

Many of these risks are mitigated in this situation because the increase in abdominal pressure displaces much of the intra-abdominal contents. Furthermore, surgical entry of the abdominal cavity for definitive management allows for evaluation and resolution of many of the possible complications. Successful placement is verified with the expulsion of air. The patient will still require definitive operative repair; thus, angiocatheter decompression of tension pneumoperitoneum should be done in consultation with a surgeon if the situation and time permits.

## CONCLUSION

Tension pneumoperitoneum has a variety of causes but ultimately requires procedural intervention. Resuscitation will likely be required prior to obtaining this diagnosis, but management will always include surgical consultation. This patient in this case is unique in that his injury was not iatrogenic or traumatic; rather he presented via EMS to the ED with spontaneous perforation after overdose. Furthermore, the needle decompression was performed in the ED prior to exploratory laparotomy. Our case illustrates use of a needle decompression technique for temporization before operative intervention that is easy to perform, provides immediate relief, and is relatively safe when done correctly.
